# Supplementary material for: A four oxidative stress gene prognostic model and integrated immunity-analysis in pancreatic adenocarcinoma
Source: Front Oncol. 2023 Jan 13;12:1015042. doi: 10.3389/fonc.2022.1015042 (PMC9880292; doi:10.3389/fonc.2022.1015042)
Supplement: Supplementary file 1 [file DataSheet_1.docx]

**Supplementary Table 1.** List of the OS genes extracted from the GeneCards with a relevance score ≥ 7.

| **Gene Symbol** | **Relevance score** | **Gene Symbol** | **Relevance score** | **Gene Symbol** | **Relevance score** |
| --- | --- | --- | --- | --- | --- |
| KIT | 7 | NDUFA10 | 9.34 | IDH1 | 13.55 |
| NDUFA13 | 7.01 | SUMO2 | 9.34 | CSF1 | 13.55 |
| MAPK13 | 7.01 | NRAS | 9.36 | DNM1L | 13.55 |
| TNFSF4 | 7.02 | IGF2BP1 | 9.36 | MET | 13.57 |
| LEPQTL1 | 7.02 | CXCL9 | 9.36 | TSC2 | 13.63 |
| MIR185 | 7.03 | SLC18A2 | 9.39 | PLA2G6 | 13.66 |
| ALS3 | 7.04 | PIK3C2A | 9.39 | PGD | 13.69 |
| ALS7 | 7.04 | BCL2L11 | 9.4 | KCNH2 | 13.69 |
| OXTR | 7.05 | STK4 | 9.41 | HMOX2 | 13.69 |
| DHFR | 7.05 | HNF4A | 9.42 | PPIF | 13.71 |
| ACACA | 7.05 | ALOX12 | 9.42 | EHHADH | 13.73 |
| GPX8 | 7.05 | GLS2 | 9.43 | TXNRD2 | 13.77 |
| BGLAP | 7.06 | CFH | 9.46 | AKR1B1 | 13.78 |
| TAZ | 7.07 | F8 | 9.47 | GSTM2 | 13.79 |
| MAPK12 | 7.08 | DNAJB1 | 9.48 | MAP2K3 | 13.79 |
| YBX1 | 7.08 | TBK1 | 9.48 | NGB | 13.8 |
| SFXN4 | 7.09 | ITIH4 | 9.49 | MMP1 | 13.81 |
| SLC4A1 | 7.09 | LRPPRC | 9.49 | NCF1 | 13.81 |
| HPSE | 7.09 | XRCC1 | 9.5 | MECP2 | 13.81 |
| RPTOR | 7.09 | TP73 | 9.5 | NPPB | 13.87 |
| MTA1 | 7.1 | ANGPT2 | 9.5 | SIRT2 | 13.9 |
| CD274 | 7.1 | AOX1 | 9.51 | STAT1 | 13.92 |
| ENDOG | 7.1 | NEAT1 | 9.51 | ECHS1 | 13.93 |
| BMP4 | 7.1 | NME1 | 9.52 | AOC3 | 13.93 |
| MTTP | 7.11 | DES | 9.53 | H6PD | 13.94 |
| TCF7L2 | 7.11 | TRPA1 | 9.54 | FGF2 | 13.94 |
| TLR6 | 7.11 | TGFA | 9.55 | GPX2 | 13.94 |
| VDR | 7.12 | MAP3K7 | 9.55 | GLRX2 | 13.95 |
| CPQ | 7.12 | EPX | 9.56 | OSER1 | 13.95 |
| NFU1 | 7.12 | PLG | 9.56 | GSTM3 | 13.97 |
| GADD45G | 7.12 | INSR | 9.56 | KRIT1 | 13.97 |
| FMO4 | 7.13 | RHOD | 9.57 | SDHAF1 | 13.99 |
| BBC3 | 7.14 | GP1BA | 9.57 | OPRM1 | 14.01 |
| AREG | 7.14 | LGALS3 | 9.58 | SESN2 | 14.05 |
| HK1 | 7.15 | CYC1 | 9.58 | APOH | 14.05 |
| PIK3R2 | 7.15 | BAG3 | 9.58 | VDAC1 | 14.06 |
| ATG5 | 7.16 | PARK12 | 9.59 | REN | 14.07 |
| POU5F1 | 7.16 | STAT4 | 9.6 | SST | 14.08 |
| SIRT6 | 7.16 | NR1H4 | 9.6 | ADRB2 | 14.09 |
| MIR214 | 7.17 | DNAH8 | 9.6 | NR3C2 | 14.11 |
| JAZF1 | 7.18 | MT-ND6 | 9.6 | GSK3B | 14.11 |
| FADD | 7.18 | NDUFA9 | 9.61 | KRAS | 14.15 |
| BCL2A1 | 7.2 | FCGR2B | 9.61 | MYC | 14.15 |
| IL11 | 7.2 | CSK | 9.62 | ADH5 | 14.15 |
| SELENOT | 7.2 | STK24 | 9.63 | HTR1A | 14.15 |
| MAP3K11 | 7.2 | AR | 9.63 | FXN | 14.16 |
| CYP4F2 | 7.21 | GYG1 | 9.64 | NPPA | 14.16 |
| MIR24-2 | 7.21 | PRKD2 | 9.69 | PTK2 | 14.19 |
| ASPA | 7.22 | PPIA | 9.69 | CPT1B | 14.19 |
| ROCK1 | 7.24 | CDK4 | 9.71 | HLA-DRA | 14.26 |
| NEDD8 | 7.26 | TPT1 | 9.71 | UGT1A1 | 14.28 |
| GAA | 7.27 | COA8 | 9.72 | PRKD1 | 14.3 |
| ATP5PD | 7.27 | IRF5 | 9.72 | CYB5R3 | 14.33 |
| PEPD | 7.28 | F5 | 9.72 | GPX4 | 14.34 |
| CAPN2 | 7.29 | SCO2 | 9.72 | GPT | 14.35 |
| PTPRC | 7.3 | PKM | 9.74 | FMO3 | 14.39 |
| CYP20A1 | 7.3 | GLO1 | 9.74 | F3 | 14.39 |
| MKI67 | 7.31 | MYH6 | 9.74 | TFRC | 14.41 |
| SORL1 | 7.31 | MME | 9.75 | JAK2 | 14.41 |
| MIR210 | 7.31 | FOXM1 | 9.75 | MT-TL1 | 14.41 |
| SORD | 7.33 | ACOX2 | 9.76 | IL1R1 | 14.41 |
| MUC5AC | 7.33 | JAK1 | 9.78 | MAP2K1 | 14.45 |
| ALDH3A2 | 7.33 | H19 | 9.78 | MT-CO2 | 14.48 |
| CHCHD2 | 7.33 | NDRG1 | 9.78 | ATP2A2 | 14.52 |
| PLD1 | 7.34 | PEX11B | 9.79 | PLA2G7 | 14.52 |
| C5 | 7.34 | ERBB2 | 9.79 | CD40 | 14.53 |
| MIR222 | 7.35 | FKBP5 | 9.82 | MAP2K4 | 14.56 |
| TNFRSF11B | 7.36 | MIR133B | 9.82 | GSTO1 | 14.56 |
| TNFRSF10A | 7.37 | TPM1 | 9.82 | TPH1 | 14.57 |
| FABP1 | 7.37 | MIR221 | 9.82 | BCL2L1 | 14.58 |
| PFKM | 7.37 | VHL | 9.83 | ACHE | 14.64 |
| PYCR2 | 7.38 | IL2RA | 9.84 | CRAT | 14.66 |
| IL6R | 7.38 | MSH2 | 9.84 | GCLC | 14.68 |
| SUMO1 | 7.39 | C4A | 9.86 | NFKBIA | 14.68 |
| TEK | 7.4 | RAB5A | 9.86 | SGCB | 14.71 |
| SLC7A1 | 7.4 | PLAUR | 9.86 | IL4 | 14.75 |
| FIG4 | 7.4 | NEIL1 | 9.87 | UCP2 | 14.77 |
| GLT8D1 | 7.4 | SPR | 9.88 | NR4A2 | 14.83 |
| LBR | 7.41 | LCK | 9.88 | EGR1 | 14.89 |
| CXCL16 | 7.41 | MSRB3 | 9.89 | IL17A | 14.88 |
| OSM | 7.42 | GLE1 | 9.89 | EGR1 | 14.89 |
| GRIN1 | 7.42 | TAF15 | 9.91 | LOC110806262 | 14.9 |
| DYRK1A | 7.42 | GFM2 | 9.92 | TRMT10C | 14.92 |
| MIR181A2 | 7.42 | SETX | 9.92 | PNPT1 | 14.92 |
| DNM2 | 7.43 | ADAMTS13 | 9.93 | CDKN2A | 14.94 |
| TACO1 | 7.43 | GHRL | 9.94 | PTK2B | 14.96 |
| GIGYF2 | 7.43 | KCNMA1 | 9.94 | CCL5 | 15.01 |
| C5AR1 | 7.44 | MIR125A | 9.95 | NPM1 | 15.05 |
| ALDH3B1 | 7.44 | H2AX | 9.96 | ETS1 | 15.05 |
| CCR7 | 7.44 | FOXP3 | 9.96 | PRODH | 15.1 |
| MUC1 | 7.45 | KRT8 | 9.97 | ACO1 | 15.13 |
| CANX | 7.45 | PLA2G2A | 9.98 | EEF1A1 | 15.17 |
| ADH1A | 7.45 | BRF2 | 9.98 | OGDH | 15.18 |
| FRZB | 7.46 | MIR200B | 9.98 | TAC1 | 15.23 |
| EEF2 | 7.47 | FKRP | 9.98 | CASP1 | 15.27 |
| ZFAND1 | 7.47 | ADPRS | 9.99 | CYP11B2 | 15.27 |
| UBQLN4 | 7.47 | H4-16 | 9.99 | NDUFAF2 | 15.28 |
| PKD1 | 7.47 | ALAD | 9.99 | PRKCD | 15.29 |
| MIR200C | 7.47 | MIR181A1 | 10 | SLC5A7 | 15.29 |
| TNIP1 | 7.48 | MT-ND4 | 10.01 | SMAD3 | 15.31 |
| SCN4B | 7.49 | NDUFB9 | 10.01 | ENG | 15.32 |
| IL16 | 7.5 | IGF2 | 10.02 | GLUL | 15.33 |
| H3C14 | 7.5 | ACTN2 | 10.04 | HSD17B4 | 15.34 |
| ARNT | 7.5 | ISCU | 10.04 | RAC2 | 15.4 |
| CYP19A1 | 7.51 | XIAP | 10.05 | MT-ND2 | 15.42 |
| SDC1 | 7.51 | CFLAR | 10.05 | PON3 | 15.42 |
| CACNA2D1 | 7.52 | GSTA2 | 10.06 | ATXN2 | 15.44 |
| AQP1 | 7.52 | NLRP3 | 10.07 | PTEN | 15.46 |
| CDC25C | 7.52 | QDPR | 10.08 | TSPO | 15.47 |
| ACTN4 | 7.53 | CASP2 | 10.1 | ELANE | 15.48 |
| BSG | 7.55 | SRF | 10.12 | SDHC | 15.49 |
| LYRM4 | 7.55 | PKLR | 10.13 | TUFM | 15.55 |
| KL | 7.56 | TYMP | 10.14 | ERN1 | 15.57 |
| ANXA11 | 7.56 | SCO1 | 10.14 | POMC | 15.59 |
| AVP | 7.56 | PTS | 10.14 | DBH | 15.61 |
| ABCC3 | 7.56 | THBS1 | 10.19 | CYP2C9 | 15.63 |
| MRAP | 7.56 | SYP | 10.2 | GPX7 | 15.63 |
| FANCD2 | 7.57 | TYK2 | 10.2 | P4HB | 15.64 |
| FIS1 | 7.57 | GRN | 10.21 | FUS | 15.69 |
| PGAM5 | 7.57 | OPTN | 10.22 | MT-ND5 | 15.7 |
| CAMKK2 | 7.57 | GRIA1 | 10.22 | FASLG | 15.7 |
| CFAP410 | 7.58 | PIK3CB | 10.22 | HLA-DRB1 | 15.74 |
| VIPR1 | 7.58 | IKBKB | 10.23 | MIR155 | 15.75 |
| ADRB3 | 7.59 | CEBPB | 10.23 | TF | 15.8 |
| RTN4 | 7.59 | CD80 | 10.23 | CHKB | 15.81 |
| MIR19A | 7.6 | C4B | 10.23 | CDK2 | 15.87 |
| FAAH | 7.6 | PPP1R15A | 10.23 | CD36 | 15.9 |
| GLA | 7.61 | ACSL4 | 10.24 | HFE | 15.91 |
| PDE4A | 7.61 | EIF2B3 | 10.24 | ACP1 | 15.95 |
| TARS2 | 7.61 | PLA2G4A | 10.25 | GSTA1 | 15.97 |
| CX3CR1 | 7.62 | TUBA1B | 10.26 | NOTCH1 | 16.03 |
| NOD2 | 7.62 | STK11 | 10.27 | DUSP1 | 16.05 |
| SFTPB | 7.62 | MMP3 | 10.27 | PRDX4 | 16.08 |
| LANCL1 | 7.63 | GRM5 | 10.29 | PTPN11 | 16.08 |
| CD38 | 7.63 | DRD3 | 10.29 | AGTR1 | 16.12 |
| FECH | 7.64 | SMARCA4 | 10.3 | HSP90B1 | 16.13 |
| TPPP3 | 7.65 | NTRK1 | 10.31 | SLC18A3 | 16.13 |
| IL1RAPL2 | 7.65 | ECE1 | 10.31 | FN1 | 16.15 |
| TP53INP1 | 7.65 | LYN | 10.32 | PRKCB | 16.18 |
| ENC1 | 7.65 | ELK1 | 10.32 | CNR1 | 16.19 |
| PEX5 | 7.66 | ALDH3A1 | 10.35 | TREM2 | 16.22 |
| IKBKG | 7.66 | PDCD1 | 10.37 | NDUFV2 | 16.23 |
| FYN | 7.66 | CCS | 10.38 | GLRX | 16.24 |
| ABCG2 | 7.66 | SIL1 | 10.39 | ITGAM | 16.27 |
| MIR199A1 | 7.67 | IL5 | 10.4 | TECRL | 16.27 |
| UCN2 | 7.67 | PSEN2 | 10.41 | IL1RN | 16.28 |
| CD46 | 7.68 | ARG2 | 10.41 | CACNA1S | 16.28 |
| KIAA0319L | 7.68 | IDO1 | 10.42 | HTT | 16.31 |
| MIR184 | 7.68 | DYNLL1 | 10.44 | PLAU | 16.33 |
| CXCL2 | 7.68 | MTR | 10.45 | TIMP1 | 16.35 |
| GPX5 | 7.69 | PDIA2 | 10.45 | CDK5 | 16.36 |
| CD28 | 7.7 | SLPI | 10.46 | CALR | 16.41 |
| YAP1 | 7.7 | SLC1A1 | 10.46 | MGST1 | 16.45 |
| GADD45B | 7.7 | EIF2B4 | 10.46 | SUOX | 16.46 |
| EIF2AK4 | 7.71 | ADAM17 | 10.48 | PTGS1 | 16.5 |
| NEK1 | 7.71 | CAMP | 10.48 | CYP2C19 | 16.58 |
| HDAC9 | 7.71 | CYP2A6 | 10.48 | TREX1 | 16.61 |
| ACAD8 | 7.71 | HDAC1 | 10.48 | TTN | 16.61 |
| MIR203A | 7.72 | CD79A | 10.52 | DLD | 16.63 |
| ADAM10 | 7.72 | CYP27A1 | 10.52 | ADCYAP1 | 16.67 |
| MT-CO3 | 7.72 | RXRA | 10.54 | CS | 16.67 |
| RAG2 | 7.73 | PTPN1 | 10.55 | TXNIP | 16.67 |
| C3 | 7.74 | EP300 | 10.57 | IL18 | 16.67 |
| HRH2 | 7.75 | CXCR3 | 10.57 | GCDH | 16.67 |
| VEGFC | 7.76 | NAT2 | 10.59 | APOA1 | 16.69 |
| SLC8A1 | 7.76 | EDNRA | 10.59 | CYGB | 16.76 |
| MRPS34 | 7.76 | PFN1 | 10.59 | CCND1 | 16.8 |
| FCGR3A | 7.77 | MT-ND3 | 10.6 | PDHA1 | 16.81 |
| IL12B | 7.78 | NTHL1 | 10.6 | ASS1 | 16.88 |
| CIITA | 7.78 | KCNJ2 | 10.61 | LRRK2 | 16.9 |
| SIAH1 | 7.79 | NRG1 | 10.62 | HTR2A | 16.92 |
| MIR23B | 7.79 | CHCHD10 | 10.63 | DRD2 | 16.94 |
| SET | 7.79 | BECN1 | 10.64 | SLC6A3 | 16.96 |
| MIR92A1 | 7.81 | CLEC4A | 10.66 | MAPKAPK2 | 17.07 |
| MMD | 7.81 | COQ2 | 10.66 | ARG1 | 17.08 |
| ITGA2 | 7.81 | EIF4EBP1 | 10.67 | GGT1 | 17.1 |
| TLR5 | 7.81 | SGK1 | 10.7 | SLC25A4 | 17.11 |
| PHYH | 7.82 | ANGPT1 | 10.7 | NPY | 17.12 |
| HSPG2 | 7.82 | SOCS3 | 10.71 | GBA | 17.2 |
| PRDM10 | 7.82 | UCP1 | 10.71 | BMP6 | 17.24 |
| RBP4 | 7.83 | MIR93 | 10.72 | HSPD1 | 17.28 |
| DYNC1H1 | 7.84 | AIF1 | 10.73 | CYP1B1 | 17.3 |
| HPRT1 | 7.85 | IL15 | 10.73 | NOX1 | 17.34 |
| SPARC | 7.85 | TRIM21 | 10.75 | HP | 17.35 |
| SELENOK | 7.85 | LTA | 10.76 | LEP | 17.37 |
| MAPK8IP1 | 7.86 | NAMPT | 10.77 | BLVRB | 17.4 |
| TFEB | 7.87 | MIR29A | 10.77 | NOS1AP | 17.42 |
| TAT | 7.88 | MFN2 | 10.77 | FARS2 | 17.5 |
| HDAC2 | 7.88 | CALM2 | 10.77 | HRAS | 17.53 |
| VNN1 | 7.88 | TIA1 | 10.78 | CALCA | 17.54 |
| MIR142 | 7.88 | RELA | 10.79 | RAC1 | 17.59 |
| GDF15 | 7.89 | NGFR | 10.79 | SNAP25 | 17.61 |
| SORCS2 | 7.89 | TRAP1 | 10.8 | PRKAA2 | 17.63 |
| MIR152 | 7.89 | HGF | 10.81 | PRKAA1 | 17.66 |
| SELENOP | 7.89 | GAP43 | 10.82 | EPO | 17.66 |
| ADSL | 7.9 | MIR433 | 10.83 | MSRB2 | 17.68 |
| NEFL | 7.9 | SLC6A2 | 10.83 | LOX | 17.68 |
| HPX | 7.91 | CD4 | 10.83 | NDUFB8 | 17.72 |
| OTC | 7.92 | MYO9A | 10.84 | PDE5A | 17.73 |
| LCAT | 7.92 | PENK | 10.86 | CRYAB | 17.74 |
| RYR3 | 7.92 | EPRS1 | 10.86 | EGFR | 17.83 |
| ISG15 | 7.93 | CDH1 | 10.87 | RPS27A | 17.83 |
| MAP3K1 | 7.93 | CFTR | 10.88 | MDM2 | 17.85 |
| BCR | 7.93 | RPS6KA5 | 10.89 | THBD | 17.87 |
| SLC25A1 | 7.94 | PVALB | 10.89 | NDUFV1 | 17.87 |
| EPHA4 | 7.94 | MRPL44 | 10.89 | CTLA4 | 17.89 |
| MAPKAPK5 | 7.95 | CTSB | 10.91 | MIR22 | 17.96 |
| ACTG1 | 7.95 | NTS | 10.91 | MAPK9 | 17.98 |
| CLIC1 | 7.97 | ERBB4 | 10.92 | NGF | 18 |
| SLC7A11 | 7.98 | EIF4G1 | 10.93 | IL2 | 18.07 |
| TLR8 | 7.98 | KLF4 | 10.93 | SRC | 18.08 |
| NCF4 | 7.98 | CYP2B6 | 10.93 | CREB1 | 18.08 |
| DGKQ | 7.99 | ACE2 | 10.94 | ATF6 | 18.21 |
| IFNAR1 | 8 | SESN1 | 10.94 | MIR146A | 18.25 |
| MMP8 | 8 | NRF1 | 10.95 | TLR2 | 18.3 |
| CDKN2B | 8 | PECAM1 | 10.95 | ATM | 18.34 |
| TOP1 | 8 | KLF2 | 10.96 | SDHD | 18.43 |
| ACO2 | 8.01 | GSS | 10.96 | IL1A | 18.48 |
| MIR25 | 8.02 | EIF2AK1 | 10.96 | NDUFS3 | 18.56 |
| CUL3 | 8.03 | FLT1 | 10.96 | MT-ATP6 | 18.57 |
| DUOX1 | 8.03 | RPS6KB1 | 10.96 | MT-CYB | 18.6 |
| CAMK4 | 8.04 | OXA1L | 10.98 | CDKN1A | 18.61 |
| CUL1 | 8.04 | ATXN3 | 10.99 | OSGIN1 | 18.63 |
| CYB5A | 8.04 | HLA-B | 11.01 | SLC1A3 | 18.66 |
| IGF2BP2 | 8.04 | ATF3 | 11.01 | ATF4 | 18.67 |
| UBE2D2 | 8.04 | RAF1 | 11.03 | GTPBP3 | 18.69 |
| FAM120A | 8.05 | MALAT1 | 11.04 | RHOA | 18.71 |
| AQP4 | 8.05 | ADA | 11.04 | PPARA | 18.75 |
| PDGFB | 8.05 | MAPK11 | 11.04 | ABL1 | 18.77 |
| MIR27A | 8.05 | GRIN2B | 11.04 | KDR | 18.82 |
| KCNE2 | 8.06 | ANXA2 | 11.06 | SPP1 | 18.82 |
| HAMP | 8.07 | COX6B1 | 11.08 | ACAD9 | 18.93 |
| BACH2 | 8.08 | HMGCL | 11.08 | AGT | 18.99 |
| PPP5C | 8.09 | CXCR4 | 11.08 | TRPM2 | 18.99 |
| AURKA | 8.1 | CXCL10 | 11.09 | GSTT1 | 19.01 |
| TLR7 | 8.1 | MMP13 | 11.09 | PIK3CA | 19.12 |
| PTPA | 8.11 | MIR122 | 11.12 | PRDX1 | 19.19 |
| WRN | 8.11 | STK39 | 11.12 | HMGB1 | 19.19 |
| TTPA | 8.12 | GCLM | 11.14 | CLU | 19.2 |
| RARA | 8.12 | TLR3 | 11.15 | PIK3CG | 19.26 |
| UCN | 8.12 | BAD | 11.15 | CDKN3 | 19.29 |
| PKP2 | 8.13 | TBP | 11.17 | HBB | 19.45 |
| KCNT1 | 8.13 | CCN2 | 11.18 | NDUFA12 | 19.49 |
| ATXN8OS | 8.13 | TJP1 | 11.18 | SELP | 19.5 |
| PARK10 | 8.13 | ALDH9A1 | 11.2 | ATP5F1A | 19.57 |
| PARK16 | 8.13 | BIRC5 | 11.2 | SERPINE1 | 19.6 |
| PARK21 | 8.13 | FCGR3B | 11.21 | C1QBP | 19.63 |
| MGMT | 8.14 | PDGFRL | 11.21 | PRDX3 | 19.65 |
| LIN28B | 8.15 | NLRP1 | 11.22 | TXNRD1 | 19.72 |
| SMPD1 | 8.15 | KCNE1 | 11.23 | SLC22A5 | 19.73 |
| GRM1 | 8.15 | GAD1 | 11.23 | MSRB1 | 19.74 |
| DMPK | 8.15 | ENO2 | 11.24 | AGER | 19.79 |
| PYCR1 | 8.16 | PEX12 | 11.25 | PRL | 19.82 |
| UTRN | 8.16 | CDKN1B | 11.26 | LDLR | 19.85 |
| MIR148B | 8.17 | GJA1 | 11.27 | CTNNB1 | 19.87 |
| FOXJ1 | 8.18 | HSPA1B | 11.27 | TRDN | 19.88 |
| MIR144 | 8.19 | LOC111365141 | 11.28 | CASQ2 | 19.93 |
| CCR5 | 8.2 | FGF1 | 11.3 | PC | 19.94 |
| SLC40A1 | 8.2 | DRD4 | 11.31 | CALM1 | 19.94 |
| ITGB3 | 8.2 | IRF1 | 11.31 | ETFB | 20.01 |
| CCL11 | 8.21 | ATF2 | 11.31 | SHC1 | 20.05 |
| MECOM | 8.21 | CDK1 | 11.33 | COMT | 20.07 |
| MIR20A | 8.21 | RNF112 | 11.33 | ANXA5 | 20.11 |
| DUSP19 | 8.22 | HMGCR | 11.34 | MMP2 | 20.24 |
| VASP | 8.22 | DHCR24 | 11.34 | TH | 20.37 |
| HAO1 | 8.22 | NTRK2 | 11.34 | SELE | 20.42 |
| MIR9-1 | 8.22 | CXCL1 | 11.35 | STAT3 | 20.45 |
| FH | 8.22 | AHSP | 11.35 | NUDT1 | 20.47 |
| CYP21A2 | 8.23 | NDUFA6 | 11.36 | MT-CO1 | 20.54 |
| CACNA1A | 8.23 | PLCG1 | 11.37 | MIR21 | 20.55 |
| BRAF | 8.23 | IRAK1 | 11.37 | EIF2S1 | 20.64 |
| NOSIP | 8.23 | FMO1 | 11.38 | EIF2AK3 | 20.7 |
| KLRK1 | 8.23 | HSP90AB1 | 11.39 | TNFRSF1A | 20.73 |
| ESR2 | 8.24 | ITGAL | 11.39 | TERT | 20.74 |
| PIK3C3 | 8.24 | AKT2 | 11.4 | IL13 | 20.85 |
| VTN | 8.24 | FDXR | 11.41 | GDNF | 20.89 |
| IL33 | 8.25 | MIR126 | 11.41 | SLC2A1 | 20.92 |
| CXCR1 | 8.25 | CCR6 | 11.42 | FAS | 20.96 |
| PRKCG | 8.25 | KIF1B | 11.43 | SOD3 | 21.02 |
| TALDO1 | 8.25 | DSPP | 11.45 | MB | 21.09 |
| DECR1 | 8.25 | CCNA2 | 11.46 | MT-ND1 | 21.17 |
| NR1H2 | 8.26 | CPOX | 11.46 | SDHA | 21.2 |
| HBEGF | 8.27 | ALDH1A1 | 11.46 | CYP1A2 | 21.25 |
| AKAP9 | 8.28 | HSPB2 | 11.47 | SDHB | 21.29 |
| MIR34C | 8.3 | CNTF | 11.47 | NR3C1 | 21.3 |
| MSR1 | 8.3 | MT3 | 11.48 | MMP9 | 21.3 |
| RPA1 | 8.31 | SDHAF2 | 11.5 | TXN2 | 21.32 |
| RCAN1 | 8.32 | EPAS1 | 11.5 | OXSR1 | 21.36 |
| FTH1 | 8.32 | LONP1 | 11.51 | HSPA8 | 21.4 |
| CYP11A1 | 8.32 | ABCC1 | 11.51 | C9orf72 | 21.45 |
| CHGA | 8.33 | SETD2 | 11.53 | GFAP | 21.51 |
| PLCB1 | 8.33 | E2F1 | 11.55 | IGF1 | 21.52 |
| SENP3 | 8.33 | MTFMT | 11.59 | TPO | 21.58 |
| RRM2B | 8.33 | CALB1 | 11.6 | EGF | 21.61 |
| PDK1 | 8.34 | MBP | 11.61 | MYH7 | 21.72 |
| SNCB | 8.34 | S100A9 | 11.62 | MSRA | 21.75 |
| MIR107 | 8.34 | NDUFS1 | 11.63 | CYP2E1 | 21.76 |
| MIR181C | 8.36 | IGF1R | 11.66 | GCH1 | 21.77 |
| CAPN3 | 8.36 | CHUK | 11.67 | ELAC2 | 21.87 |
| RNASE3 | 8.36 | DNASE1 | 11.67 | MAOB | 21.96 |
| PRPH | 8.37 | IFNB1 | 11.7 | PRDX6 | 22.03 |
| CFI | 8.37 | VIM | 11.7 | CYP1A1 | 22.34 |
| LAMP1 | 8.39 | ANK2 | 11.72 | PINK1 | 22.38 |
| BLOC1S1 | 8.39 | MAP2K7 | 11.73 | GPX3 | 22.42 |
| PML | 8.39 | CR2 | 11.74 | ACADL | 22.44 |
| CCK | 8.39 | DLG4 | 11.75 | SP1 | 22.49 |
| TIMP2 | 8.4 | DRD1 | 11.76 | NOX4 | 22.67 |
| TRAF2 | 8.41 | PCNA | 11.77 | CASP9 | 22.79 |
| STIP1 | 8.42 | ADCY10 | 11.78 | F2 | 22.83 |
| NOSTRIN | 8.42 | PLAT | 11.79 | ETFA | 22.88 |
| MYLK | 8.43 | TSC1 | 11.8 | PPARG | 22.88 |
| GSTM5 | 8.43 | ELN | 11.81 | HTRA2 | 22.97 |
| TPK1 | 8.43 | MBL2 | 11.82 | ADIPOQ | 23 |
| SLC2A4 | 8.43 | BMP2 | 11.83 | CYP3A4 | 23.06 |
| HLA-A | 8.44 | LCN2 | 11.85 | ALDH2 | 23.14 |
| UNG | 8.44 | GLUD1 | 11.86 | FOXO3 | 23.38 |
| IL3 | 8.46 | TNFSF10 | 11.88 | COX5A | 23.46 |
| RORA | 8.46 | DMD | 11.89 | SELENON | 23.56 |
| MDH1 | 8.46 | MCL1 | 11.89 | OGG1 | 23.62 |
| RUNX2 | 8.46 | NOL3 | 11.9 | KNG1 | 23.67 |
| SCARA3 | 8.46 | CST3 | 11.91 | MTOR | 23.71 |
| PDIA3 | 8.47 | CBS | 11.92 | CHAT | 23.77 |
| DDC | 8.47 | NDUFS8 | 11.92 | ABCD1 | 23.89 |
| GSTM4 | 8.47 | CHKA | 11.96 | MTO1 | 24.03 |
| PIGA | 8.48 | DDAH2 | 11.99 | TLR4 | 24.05 |
| BRCA2 | 8.48 | NTF3 | 11.99 | BAX | 24.15 |
| UNC13A | 8.49 | ACTB | 12 | PRDX2 | 24.22 |
| VPS13C | 8.49 | PRKAB1 | 12 | POLG | 24.4 |
| TNFSF11 | 8.49 | STUB1 | 12.02 | MAPK3 | 24.43 |
| CYP2C8 | 8.5 | FCGR2A | 12.02 | VCAM1 | 24.45 |
| SERPINF1 | 8.5 | ITGB2 | 12.04 | HSF1 | 24.45 |
| DYSF | 8.51 | MATR3 | 12.05 | NCF2 | 24.62 |
| PSIP1 | 8.51 | APOB | 12.06 | PRNP | 24.79 |
| TPI1 | 8.52 | STK25 | 12.06 | FOS | 24.94 |
| PDGFRB | 8.52 | TLR9 | 12.06 | MAOA | 25.05 |
| CAMK2G | 8.52 | CSF2 | 12.07 | APOE | 25.05 |
| EPHX2 | 8.53 | APAF1 | 12.08 | HIF1A | 25.17 |
| CR1 | 8.53 | PDYN | 12.08 | CACNA1C | 25.18 |
| CYP3A5 | 8.54 | OPRD1 | 12.09 | CASP8 | 25.25 |
| AGRN | 8.54 | TNFAIP3 | 12.09 | LMNA | 25.28 |
| NR2C2 | 8.57 | ABCB1 | 12.09 | XBP1 | 25.28 |
| XRCC6 | 8.58 | TGM2 | 12.1 | CRH | 25.53 |
| PPP3CA | 8.59 | ACTA1 | 12.12 | PPARGC1A | 25.65 |
| UBQLN1 | 8.59 | MIR23A | 12.14 | CAV1 | 26.16 |
| FOXO4 | 8.6 | GRB2 | 12.17 | PON2 | 26.18 |
| MAPK7 | 8.6 | HSD17B10 | 12.21 | BCL2 | 26.23 |
| NES | 8.6 | MRPS14 | 12.23 | TARDBP | 26.28 |
| FTL | 8.6 | MIR132 | 12.24 | MAPK10 | 26.31 |
| BACH1 | 8.61 | LTF | 12.26 | HSPA1A | 26.33 |
| SCGB1A1 | 8.61 | CCL4 | 12.26 | ACE | 26.46 |
| FMO2 | 8.61 | NDUFS2 | 12.26 | APEX1 | 26.49 |
| APC | 8.63 | BTD | 12.26 | OLR1 | 26.49 |
| MYD88 | 8.63 | IFNA1 | 12.26 | ESR1 | 26.5 |
| HERPUD1 | 8.64 | RB1 | 12.27 | MAP3K5 | 26.53 |
| CYP17A1 | 8.64 | SMAD4 | 12.27 | VCP | 26.6 |
| TYRP1 | 8.65 | MAP2K6 | 12.31 | AIFM1 | 26.64 |
| XRCC5 | 8.65 | SRXN1 | 12.31 | ICAM1 | 26.7 |
| FKBP1B | 8.66 | LPO | 12.31 | CP | 26.94 |
| MMP14 | 8.66 | HBA1 | 12.32 | SCN5A | 26.98 |
| TMEM161A | 8.67 | IRS1 | 12.32 | HSPB1 | 27.11 |
| ALOX15 | 8.68 | SLC17A5 | 12.34 | BDNF | 27.3 |
| SOX2 | 8.68 | KCNQ1 | 12.34 | NFKB1 | 27.4 |
| ASL | 8.68 | HTR2C | 12.35 | PRDX5 | 27.52 |
| CCNF | 8.68 | ENO1 | 12.36 | HBG2 | 27.57 |
| DIABLO | 8.69 | LAMP2 | 12.37 | CAV3 | 27.57 |
| MMP7 | 8.69 | ITGB1 | 12.37 | TYR | 27.6 |
| TRPV4 | 8.7 | SERPINA1 | 12.37 | KEAP1 | 27.7 |
| PGK1 | 8.7 | S100B | 12.37 | GSTM1 | 27.84 |
| NDUFA1 | 8.7 | LOC110973015 | 12.37 | AARS2 | 27.95 |
| IL23A | 8.7 | REST | 12.4 | FOXO1 | 27.96 |
| ATXN1 | 8.71 | PIK3R1 | 12.4 | PSEN1 | 27.99 |
| JUNB | 8.71 | CDC42 | 12.4 | DDIT3 | 28.04 |
| HSPA14 | 8.71 | CXCL12 | 12.4 | GSTP1 | 28.09 |
| CD86 | 8.72 | DAO | 12.41 | CPT1A | 28.1 |
| GAL | 8.72 | BAK1 | 12.41 | SLC25A20 | 28.34 |
| FGF7 | 8.73 | HCRT | 12.41 | GPX1 | 28.43 |
| SCP2 | 8.73 | ADORA2A | 12.43 | SQSTM1 | 28.47 |
| BCL6 | 8.73 | MTHFR | 12.46 | ETFDH | 28.53 |
| SULT1A3 | 8.74 | CSF3 | 12.47 | HSPA5 | 28.54 |
| GADD45A | 8.74 | BRCA1 | 12.47 | ACOX1 | 28.56 |
| GSN | 8.75 | GNAS | 12.48 | CYP2D6 | 28.87 |
| MDH2 | 8.75 | SIGMAR1 | 12.48 | VWF | 28.87 |
| IL2RB | 8.76 | IREB2 | 12.5 | NDUFS4 | 28.94 |
| ABCC8 | 8.76 | TGFBR1 | 12.5 | GAPDH | 29.13 |
| PPIG | 8.77 | CARS2 | 12.51 | MAPT | 29.16 |
| MCU | 8.77 | CALM3 | 12.52 | PARP1 | 29.43 |
| MLYCD | 8.78 | MAP2 | 12.56 | VEGFA | 29.48 |
| CHMP2B | 8.78 | MIF | 12.56 | RYR2 | 29.56 |
| UBC | 8.79 | CTSD | 12.56 | CCL2 | 29.85 |
| MSN | 8.79 | MAPKAPK3 | 12.58 | HADHB | 29.98 |
| PTPN3 | 8.79 | PAH | 12.59 | RYR1 | 30.4 |
| NTF4 | 8.8 | OXR1 | 12.6 | CYBB | 30.6 |
| SLC19A3 | 8.8 | CACNB4 | 12.61 | GFM1 | 30.79 |
| CCNB1 | 8.8 | PXN | 12.61 | HSPA4 | 31.1 |
| FMR1 | 8.8 | HTR3A | 12.62 | PTGS2 | 31.12 |
| HSPA6 | 8.81 | IGF2R | 12.62 | SNCA | 31.58 |
| HK2 | 8.82 | CASP7 | 12.65 | IFNG | 31.62 |
| SELL | 8.83 | GSTO2 | 12.66 | NQO1 | 31.87 |
| PRKCZ | 8.83 | ANG | 12.66 | HSP90AA1 | 31.95 |
| PTGIS | 8.85 | EPHA3 | 12.66 | EDN1 | 32.13 |
| ZC3H12A | 8.85 | PTPN22 | 12.67 | TGFB1 | 32.32 |
| AHR | 8.87 | MRPS16 | 12.67 | JUN | 32.45 |
| GLS | 8.87 | GLUD2 | 12.74 | CYBA | 32.63 |
| PPOX | 8.87 | CREBBP | 12.75 | CXCL8 | 32.79 |
| NCAM1 | 8.88 | SLC25A13 | 12.75 | CRP | 33.11 |
| RETN | 8.88 | SIRT3 | 12.75 | ALB | 33.45 |
| B2M | 8.89 | GSTA4 | 12.77 | SIRT1 | 33.85 |
| CD55 | 8.89 | IDH2 | 12.79 | ACADS | 33.91 |
| VKORC1L1 | 8.89 | TOR1A | 12.84 | HADH | 34.03 |
| SLC25A27 | 8.89 | DCTN1 | 12.84 | AKT1 | 34.11 |
| EDNRB | 8.9 | MIR17 | 12.86 | INS | 34.82 |
| PF4 | 8.91 | C12orf65 | 12.86 | IL10 | 35.03 |
| SMAD2 | 8.91 | EPHX1 | 12.88 | ACADVL | 35.49 |
| SOCS1 | 8.91 | TGFBR2 | 12.88 | TXN | 35.54 |
| BLK | 8.94 | BCHE | 12.91 | CASP3 | 35.93 |
| PYGM | 8.94 | ERCC6 | 12.93 | G6PD | 36.15 |
| CDK6 | 8.95 | NFE2L1 | 12.93 | MAPK1 | 36.3 |
| MICB | 8.95 | EIF2AK2 | 12.94 | SLC6A4 | 36.52 |
| PPARD | 8.96 | ADM | 12.95 | ACADM | 37.37 |
| UBQLN2 | 8.99 | H2AC18 | 12.96 | IL1B | 37.44 |
| HYOU1 | 9 | COL2A1 | 12.98 | CYCS | 38.24 |
| TACR1 | 9.01 | TRPV1 | 12.99 | MAPK8 | 38.51 |
| BDKRB2 | 9.01 | VIP | 13 | HADHA | 38.55 |
| ADRB1 | 9.02 | SYK | 13 | PRKN | 38.63 |
| CTTN | 9.03 | A2M | 13.01 | IL6 | 38.95 |
| DEPDC5 | 9.03 | ALOX5 | 13.01 | PON1 | 40.21 |
| MIR106B | 9.04 | CD44 | 13.02 | PARK7 | 40.56 |
| NQO2 | 9.04 | IAPP | 13.02 | GSR | 42.22 |
| ABCC2 | 9.04 | PRKCA | 13.03 | XDH | 43.46 |
| SREBF1 | 9.05 | SNCAIP | 13.04 | APP | 43.81 |
| SERPINH1 | 9.05 | S100A8 | 13.04 | MAPK14 | 44.95 |
| H2BC21 | 9.06 | NAGS | 13.04 | MPO | 45.08 |
| LPA | 9.07 | HNRNPA1 | 13.04 | SOD2 | 48.82 |
| VCL | 9.07 | ATP13A2 | 13.05 | CPT2 | 49.36 |
| MIR145 | 9.08 | SLC25A3 | 13.07 | NFE2L2 | 54.46 |
| MIR143 | 9.09 | DAPK1 | 13.08 | TP53 | 57.5 |
| FASN | 9.1 | TTR | 13.09 | NOS1 | 58.58 |
| CASP4 | 9.1 | EIF4E | 13.11 | HMOX1 | 60.47 |
| CD34 | 9.11 | DLST | 13.14 | TNF | 63.9 |
| KRT18 | 9.11 | CRYAA | 13.14 | CAT | 70.77 |
| CTSG | 9.12 | UBE2L3 | 13.16 | NOS2 | 71.08 |
| SLC11A2 | 9.12 | NEFH | 13.16 | SOD1 | 80.41 |
| ODC1 | 9.12 | G3BP1 | 13.17 | NOS3 | 82.97 |
| TNFRSF10B | 9.13 | ADH1C | 13.21 |  |  |
| CHEK1 | 9.13 | DNMT1 | 13.21 |  |  |
| SFTPD | 9.13 | MRPS22 | 13.23 |  |  |
| CD69 | 9.14 | TFAM | 13.24 |  |  |
| PDLIM4 | 9.14 | OSGIN2 | 13.24 |  |  |
| GH1 | 9.14 | CCL3 | 13.25 |  |  |
| LPL | 9.14 | SCN4A | 13.26 |  |  |
| HNF1A | 9.14 | UCHL1 | 13.26 |  |  |
| SCARB1 | 9.15 | MIR195 | 13.27 |  |  |
| PTX3 | 9.15 | FGFR1 | 13.29 |  |  |
| RAD51 | 9.16 | OXT | 13.3 |  |  |
| EZH2 | 9.16 | MIR34A | 13.32 |  |  |
| COX15 | 9.16 | AKR1A1 | 13.34 |  |  |
| LGALS1 | 9.17 | OPA1 | 13.34 |  |  |
| GRIN2A | 9.19 | VARS2 | 13.34 |  |  |
| VAPB | 9.2 | SLC1A2 | 13.35 |  |  |
| ELAVL1 | 9.2 | CRHR1 | 13.36 |  |  |
| HRH1 | 9.22 | POR | 13.37 |  |  |
| ATR | 9.23 | PRKG1 | 13.37 |  |  |
| PNKP | 9.24 | TGFB3 | 13.37 |  |  |
| GFER | 9.24 | KCNJ5 | 13.37 |  |  |
| CDH5 | 9.25 | BACE1 | 13.39 |  |  |
| MIR24-1 | 9.25 | SNTA1 | 13.39 |  |  |
| ABCA1 | 9.25 | DRD5 | 13.41 |  |  |
| DAXX | 9.25 | SERPINA3 | 13.41 |  |  |
| CDH2 | 9.26 | HSPA9 | 13.41 |  |  |
| GZMB | 9.27 | SCN2A | 13.42 |  |  |
| CALB2 | 9.28 | AMPD1 | 13.44 |  |  |
| MUTYH | 9.29 | TNFRSF1B | 13.47 |  |  |
| ERCC8 | 9.29 | ITPR1 | 13.47 |  |  |
| DDAH1 | 9.3 | TSFM | 13.47 |  |  |
| NDUFS7 | 9.3 | MT-TK | 13.49 |  |  |
| NDUFS6 | 9.31 | MIR223 | 13.49 |  |  |
| ERO1A | 9.32 | TGFB2 | 13.52 |  |  |
| DSP | 9.32 | UCP3 | 13.54 |  |  |

**Supplementary Table 2.** List of immune checkpoint related genes

| **Gene Symbol** | **Gene Symbol** | **Gene Symbol** | **Gene Symbol** |
| --- | --- | --- | --- |
| TNFSF14 | CD96 | HLA-DRB1 | HLA-DPB1 |
| TNFSF15 | HLA-A | TNFSF9 | HLA-DMB |
| CD40LG | SIRPA | BTN2A2 | HLA-DMA |
| CD276 | CD47 | HLA-DOA | CD160 |
| TNFSF18 | TNFRSF9 | TNFRSF18 | CD70 |
| HLA-DRB5 | CD28 | HLA-C | BTNL9 |
| TDO2 | CD48 | HLA-DPA1 | HLA-DRA |
| HLA-DQB1 | BTLA | PVR | CD200R1 |
| KIR2DL4 | TNFSF4 | BTN2A1 | HLA-DOB |
| CD228 | CD27 | CD244 | IDO2 |

**Supplementary Table 3.** List of synthetic driver genes of T cell proliferation

| **Gene Symbol** | **Gene Symbol** | **Gene Symbol** | **Gene Symbol** |
| --- | --- | --- | --- |
| IFNL2 | CDK2 | LIG3 | AHNAK |
| LTBR | MS4A3 | ZNF830 | ADA |
| IL1RN | CDK1 | DCLRE1B | MRPL18 |
| CXCL12 | DBI | SLC10A7 |  |
| CRLF2 | CYP27A1 | CALML3 |  |
| IL12B | AKR1C4 | CLIC1 |  |
| NFYB | DUPD1 | RAN |  |
| BATF | GPD1 | ITM2A |  |
| FOSB | GPN3 | HOMER1 |  |
| ATF6B | AHCY | MRPL51 |  |
